# Bifactor Modeling of the Social Interaction Anxiety Scale-6 and the Social Phobia Scale-6 in a Korean Community Sample

**DOI:** 10.31083/AP46107

**Published:** 2025-08-28

**Authors:** Young-Jin Lim

**Affiliations:** ^1^Department of Psychology, Gachon University, 13120 Seongnam, Republic of Korea

**Keywords:** SIAS-6, SPS-6, factor analysis, Korean

## Abstract

**Background:**

The Social Interaction Anxiety Scale-6 (SIAS-6) and Social Phobia Scale-6 (SPS-6) are self-reported measures of social anxiety. The aim of this study was to identify the best model for SIAS-6 and SPS-6 using the newly advanced method of exploratory structural equation modeling (ESEM).

**Methods:**

Both confirmatory factor analysis (CFA) and ESEM were utilized to assess the factor structure of the SIAS-6 and SPS-6. Three hundred Korean adults (*n*_female_ = 150, aged: 39.28 ± 10.91 years) participated in an online survey and responded to the SIAS-6 and SPS-6 questionnaires.

**Results:**

The findings showed that the bifactor ESEM and bifactor CFA models were a better fit than the other models. General factors had high loading values and reliability coefficients, whereas specific factors had moderate loading values and reliability coefficients. Additionally, measurement invariance across sexes was established.

**Conclusion:**

This study demonstrated that bifactor models provide a unified perspective on the varying viewpoints regarding the relationship between social interaction and social performance anxiety.

## Main Points

1. The bifactor models of the SIAS-6 and SPS-6 had a better model fit than the other models.

2. Social interaction and social performance anxiety exist as a shared concept under the name of a general factor, but at the same time, there are separate concepts for each.

3. The bifactor models of the SIAS-6 and SPS-6 had measurement invariance.

## 1. Introduction

Social anxiety refers to the fear of evaluation by other people in social or performance situations. When the severity of social anxiety increases and causes a decline in a person’s functioning, it is called social anxiety disorder [1]. Social anxiety disorder is one of the most common types of anxiety disorders, with a lifetime prevalence rate of 4.0–12.1% [2, 3, 4]. Social anxiety disorder has been reported to deteriorate an individual’s academic, work, and interpersonal functioning while also reducing emotional well-being [5, 6, 7]. Social anxiety can be divided into social interaction anxiety and social performance anxiety [8].

Several scales have been developed to assess social anxiety [9]. Among them, the most used tools include the Social Interaction Anxiety Scale (SIAS) and the Social Phobia Scale (SPS) [10]. The SIAS measures social interaction anxiety, defined as the fear and avoidance experienced in social situations, whereas the SPS measures social performance anxiety, which refers to the degree to which one fears being scrutinized in performance contexts. As these two scales measure two distinct aspects of social anxiety, they are often used together.

Two shortcomings of these two scales were identified. Specifically, the number of items in these scales was too large, which could have caused respondent fatigue, and the goodness-of-fit index of the factor analysis was relatively low [11, 12]. To solve these problems, short forms of these two scales, the Social Interaction Anxiety Scale-6 (SIAS-6) and Social Phobia Scale-6 (SPS-6), were developed [13].

These two short forms have been reported to have sound psychometric properties and have been validated in various cultures [14, 15, 16, 17]. However, there is still no consensus on the factor structures of the SIAS-6 and SPS-6. Some studies have reported a two-factor structure in which items belonging to the SIAS-6 constitute one factor and items belonging to the SPS-6 constitute another factor [13, 14]. However, other studies reported that the SIAS-6 and SPS-6 showed a bifactor model in which all 12 items loaded onto a single general social anxiety factor [18, 19].

To resolve this discrepancy, this study used newly developed exploratory structural equation modeling (ESEM) to scrutinize the factor structures of the SIAS-6 and SPS-6. ESEM complements the shortcomings of the previously used exploratory factor analysis (EFA) and confirmatory factor analysis (CFA) [19]. For example, ESEM compensates for the shortcomings of CFA by allowing cross-loadings between factors and compensates for the shortcomings of EFA by specifying the relationship between items and factors in the prior.

ESEM is also known to solve the CFA problem of the overestimation of correlations between factors [20]. The reason the correlation between factors in the ESEM is relatively small is that the ESEM allows items to load on multiple factors, which leads to more accurate and realistic factor correlations. CFA’s stricter factor structure, which does not allow items to load onto multiple factors, tends to inflate these correlations [20].

To date, no study has explored the factor structure of the SIAS-6 and SPS-6 using ESEM. Therefore, this study aimed to identify the most appropriate model for the SIAS-6 and SPS-6 through ESEM in a non-clinical Korean population. The specific objectives of this study are as follows: (1) to determine the best model for the SIAS-6 and SPS-6 using both ESEM and CFA techniques; (2) to assess the internal consistency of this optimal model; and (3) to examine the measurement invariance of the best model across genders.

## 2. Methods

### 2.1 Participants

A total of 300 Korean adults (150 female) from a large online panel using quotas for age, gender, and region of residence participated in this study. We followed the suggestions of Bentler and Chou [21] to determine the appropriate sample size. They suggested a 5:1 ratio of sample size to parameters. This proposal required a minimum of 270 participants. In this study, 300 participants were selected to compensate for missing data. To prevent biased sampling, the numbers of participants were equalized by age, gender, and region. Equal numbers of participants were aged 20–29 years, 30–39 years, 40–49 years, and 50–59 years. In addition, the number of participants by region was proportional to the population, and the gender ratio of the participants was 1:1. The average age of the 300 study participants was 39.28 years (standard deviation (SD) = 10.91), and the age range was 20–59 years. Data were collected using Embrain (https://www.embrain.com/kor/), an online survey company. We collected information about the participants’ gender, age, and living region, but did not collect any identifiable personal information. Participants were provided with information about the survey’s purpose prior to the survey, agreed to confidentiality, and received points that could be used at the survey site in return for their participation.

### 2.2 Measures

#### 2.2.1 Social Interaction Anxiety Scale-6 (SIAS-6)

SIAS-6 is a self-reported short form created by Peters *et al*. [13] based on the original 20-item SIAS, which is a scale of social interaction anxiety characterized by distress experienced during interpersonal and social encounters [10]. The SIAS-6 is made up of six items. Participants respond to each item on a scale ranging from 0 = *not at all* to 4 = *extremely*. The Korean version of the SIAS-6 was used [22]. Cronbach’s alpha and McDonald’s omega were both 0.89 in the present study. While the former is calculated based on inter-item correlations, the latter is based on factor analysis results. In this study, the two internal consistency values, Cronbach’s alpha and McDonald’s omega, for the SIAS-6 were similar. This indicated that a single latent factor or construct explained most of the variance in the six items of the SIAS-6. Additionally, for the SIAS-6, there are no individual items with unequal factor loadings, which may cause Cronbach’s alpha to be lower than McDonald’s omega [23].

#### 2.2.2 Social Phobia Scale-6 (SPS-6)

The SPS-6 is a six-item scale designed to assess social performance anxiety, which refers to the fear of being negatively evaluated in particular performance contexts [10]. The scale was developed by Peters *et al*. [13] based on the original 20-item scale of the SPS [10]. Participants respond to each item on a scale from 0 (“not at all”) to 4 (“extremely”). This study utilized the Korean version of the SPS-6 [22]. Cronbach’s alpha and McDonald’s omega in the present study were reported as 0.91 and 0.90, respectively. In this study, the two internal consistency values were similar. This shows that the SPS-6 is a scale measuring a single latent factor, social performance anxiety. Additionally, it indicates that no individual item on the SPS-6 is problematic, such as having excessively low correlations with the overall score of the SPS-6, which may cause Cronbach’s alpha to be underestimated compared to McDonald’s omega [23].

### 2.3 Data Analyses

Data analyses were conducted using SPSS version 27 (IBM, Armonk, NY, USA) and Mplus version 8.8 (Muthén & Muthén, Los Angeles, CA, USA), with a *p*-value of less than 0.05 deemed statistically significant. Descriptive statistical analysis was performed using SPSS version 27, and factor analysis, reliability assessment, and measurement invariance tests were conducted using Mplus version 8.8. The factor structures of the SIAS-6 and SPS-6 were examined using CFA and ESEM, with ESEM performed using oblique target rotation. Target rotation guides the rotation of factor loadings based on a predefined target matrix, which was specified by the researcher to indicate the expected pattern of loadings. In this matrix, some elements are set to zero or near zero, reflecting the expectation that certain factors will have no or minimal loadings on specific items [19, 20].

Table 1 presents the cut-off criteria for the goodness-of-fit index used in this study [24]. The models were compared according to the fit indices listed in Table 1:

**Table 1.** **Cut-off criteria for Goodness-of-fit indices**.

Goodness-of-fit index	Good fitting	Acceptable fitting
relative/normed chi-squared (χ^2^*/df*)	0.00 ≤ χ^2^*/df * ≤ 2.00	2.00 < χ^2^*/df* ≤ 3.00
comparative fit index (CFI)	0.97 ≤ CFI < 1.00	0.95 ≤ CFI < 0.97
tucker-lewis index (TLI)	0.97 ≤ TLI < 1.00	0.95 ≤ TLI < 0.97
standardized root mean square residual (SRMR)	0.00 ≤ SRMR ≤ 0.05	0.05 < SRMR ≤ 0.10
root mean square error of approximation (RMSEA)	0.00 ≤ RMSEA ≤ 0.05	0.05 < RMSEA ≤ 0.08

Note: χ^2^, Chi-squared goodness of fit test; *df*, degrees of freedom.

(1) **Model 1:** A one-factor model in which all 12 items load on the single factor of global social anxiety.

(2) **Model 2**: A two-factor CFA model in which six items load on the social interaction anxiety factor, while the remaining six items load on the social performance anxiety factor.

(3) **Model 3**: A bifactor CFA model in which all 12 items load on the general factor (global social anxiety factor) and, at the same time, on one of the specific factors (either the social interaction anxiety factor or the social performance anxiety factor).

(4) **Model 4**: A two-factor ESEM model in which all 12 items load on the social interaction anxiety factor and the social performance anxiety factor.

(5) **Model 5**: A bifactor ESEM model in which all 12 items load on the general factor of global social anxiety while simultaneously loading on the specific factors of social interaction anxiety and social performance anxiety.

In addition, the Akaike Information Criterion (AIC) was calculated to compare the fitness of several models and determine the most appropriate model. The AIC addresses the risk of overfitting and underfitting by trading off simplicity and fit of the model. If the difference in the AIC between the two models is six or more, the model with the smaller AIC is considered to have a better fit than the model with the larger AIC [25].

In the comparison of the bifactor models, the average and lowest loading values for the designated factor were calculated along with the fit indices. The average loading values of the designated factor must be greater than 0.50 and the lowest loading values of the designated factor must be greater than 0.30 for a well-defined factor [26]. Coefficient omega hierarchical (ω_H_) was calculated as an estimate of the general factor, while coefficient omega hierarchical subscale (ω_H⁢S_) was calculated as an estimate of the specific factors to assess the internal consistency coefficients of the models that exhibited the best fit. This is because the omega coefficient is more suitable than the traditional Cronbach’s alpha for measuring the internal consistency of multidimensional constructs [23]. ω_H_ denotes the variance attributable to the general factor after controlling for all specific factors and ω_H⁢S_ is the variance attributable to the specific factor after controlling for the general factor. The acceptable cut-off score of ω_H_ is 0.50 [27]. The substantial cut-off score of ω_H⁢S_ is above 0.30, the moderate cut-off score is above 0.20 and below 0.29, and the low cut-off score is below 0.19 [28]. Additionally, the explained common variance (ECV) was calculated for the bifactor models. The ECV associated with the general factor represents the share of total common variance explained by that factor, while the ECV linked to the specific factor shows the variance uniquely tied to that specific factor. This distinction allows for a clearer understanding of how much of the variance in the data can be explained by overarching constructs (the general factor) compared with how much is uniquely explained by individual factors (the specific factor). If a general factor explains more than 70% of the common variance, the measure is considered to have a unidimensional structure, rather than multiple distinct factors [29].

Measurement invariance was evaluated using Chen’s criteria [30]. Chen [30] suggests a criterion of –0.01 for changes in comparative fit index (CFI) and a criterion of 0.015 for changes in root mean square error of approximation (RMSEA). The fit indices of each model were compared. The metric invariance model, where loadings were constrained to be equal across genders, was compared with the configural invariance model, where structural parameters were constrained to be equal across genders. A scalar invariance model, where the intercept and measurement residuals were constrained to be invariant across genders, was compared with a configural invariance model.

Two-sided multivariate tests were conducted to assess the fit of skewness and kurtosis in verifying multivariate normality for CFA and ESEM. These tests examine the properties of two distribution curves: multivariate skewness and multivariate kurtosis. Results from these tests that do not support multivariate normality may call into question the validity of subsequent CFA and ESEM analyses.

## 3. Results

### 3.1 Factor Analysis

Drawing on theory and previous studies, we compared five competing models: the one-factor model, two-factor CFA model, bifactor CFA model, two-factor ESEM model, and bifactor ESEM model. Prior to this, we conducted two-sided multivariate tests for skewness and kurtosis. The tests showed that the data deviated from multivariate normality (two-sided multivariate test for skewness fit = 31.97, *p* < 0.001; two-sided multivariate test for kurtosis fit = 244.02, *p* < 0.001). Consequently, a robust maximum likelihood estimator was employed to address non-normality. A robust maximum likelihood estimator can deliver reliable estimates even when the data fail to satisfy the usual assumptions of normality. Table 2 reports the model fit indices of the five different models. The 90% confidence interval (CI) for the RMSEA represents a range of plausible values for the population RMSEA. For the one-factor model and the two-factor CFA model, χ^2^*/df*, CFI, TLI, and RMSEA showed a poor model fit and the SRMR displayed an acceptable model fit to the data. For the bifactor CFA model, χ^2^*/df*, TLI, and RMSEA exhibited an acceptable fit and the CFI and SRMR indices indicated a good fit. For the two-factor ESEM model, TLI indices showed a poor model fit to the data; χ^2^*/df*, CFI, and RMSEA displayed an acceptable model fit; and the SRMR indicated a good model fit. For the bifactor ESEM model, both TLI and RMSEA values suggested an acceptable model fit and the CFI, SRMR, and χ^2^*/df* indicated a good fit to the data.

**Table 2.** **Goodness-of-fit tests of the competing models**.

Model	χ ^2^	*df*	χ ^2^ */df*	CFI	TLI	SRMR	RMSEA	90% CI	AIC
Model 1	461.06	54	8.54	0.826	0.787	0.071	0.159	0.145–0.172	9032.475
Model 2	224.37	53	4.23	0.927	0.909	0.057	0.104	0.090–0.118	8797.782
Model 3	92.99	41	2.27	0.978	0.964	0.025	0.065	0.048–0.083	8690.400
Model 4	100.13	43	2.33	0.961	0.940	0.028	0.067	0.050–0.084	8734.182
Model 5	60.51	33	1.83	0.981	0.962	0.019	0.053	0.031–0.073	8693.182

Note: χ^2^, Chi-squared statistic; *df*, degrees of freedom; 90% CI, 90% confidence interval; AIC, Akaike information criteria; Model 1, one-factor model; Model 2, two-factor confirmatory factor analysis (CFA) model; Model 3, bifactor CFA model; Model 4, two-factor exploratory structural equation modeling (ESEM) model; Model 5, bifactor ESEM model.

The relative model fit, which refers to determining which of two or more models best represents the data, was assessed using the AIC. Model 2 was a better fit than Model 1 (ΔAIC = –234.70). Model 4 was a better fit than Model 2 (ΔAIC = –63.60). Model 3 was a better fit than Model 4 (ΔAIC = –43.78). Additionally, Model 5 was superior to Model 4 (ΔAIC = –41.00). Thus, the bifactor CFA and ESEM models were the best alternatives.

The standardized loadings from the bifactor CFA models of the SIAS-6 and SPS-6 are listed in Table 3. In the bifactor CFA model, the average loading values on the general factor were above 0.50 (λ = 0.576–0.848, *M* = 0.662). However, the average loading values were below 0.50 and the lowest loading values were below 0.30 on social interaction anxiety (λ = 0.121–0.651, *M* = 0.426) and social performance anxiety (λ = 0.018–0.711, *M* = 0.399) factors. This suggests that each item had stronger loadings on the general factor compared with their specific factors. For the social interaction anxiety factor, five social interaction anxiety items (items 1, 2, 3, 4, and 5) loaded significantly onto the social interaction anxiety factor (λ ≥0.30), whereas only one social interaction anxiety item (item 6) failed to load onto the specific factor (λ = 0.121). Five social performance anxiety items (items 7, 9, 10, 11, and 12) loaded significantly onto the social performance anxiety factor (λ ≥0.30), whereas only one social performance anxiety item (item 8) failed to load onto the specific factor (λ = –0.018). That is, out of the 12 items, 10 items loaded significantly onto their respective specific factors, suggesting that specific factor cross-loadings remained in the bifactor CFA model even after controlling for the general factor.

**Table 3.** **Standardized factor loadings for the bifactor CFA outcomes of the SIAS-6 and SPS-6**.

Item	General Factor	Factor I	Factor II
1	**0.647**	**0.417**	0
2	**0.576**	**0.651**	0
3	**0.664**	**0.409**	0
4	**0.618**	**0.446**	0
5	**0.596**	**0.511**	0
6	**0.674**	**0.121**	0
7	**0.743**	0	**0.308**
8	**0.848**	0	**–0.018**
9	**0.699**	0	**0.364**
10	**0.654**	0	**0.425**
11	**0.612**	0	**0.711**
12	**0.609**	0	**0.566**
*ω*_H_	0.795		
*ω*_H⁢S_		0.282	0.224
ECV	0.682	0.159	0.158

Note: Target loadings are in bold font. Factor I, social interaction anxiety; Factor II, social performance anxiety; ω_H_, coefficient omega hierarchical; ω_H⁢S_, coefficient omega hierarchical subscale; ECV, explained common variance; SIAS-6, the Social Interaction Anxiety Scale-6; SPS-6, Social Phobia Scale-6.

Table 4 shows the standardized loadings from the bifactor ESEM models of the SIAS-6 and SPS-6. All items showed significant loadings for the general factors. In the bifactor ESEM, the average loadings on the general factor were above 0.50 (λ = 0.536–0.905, *M* = 0.659). However, the average loadings were below 0.50 and the lowest loadings were below 0.30 for the social interaction anxiety (λ = 0.157–0.644, *M* = 0.444) and social performance anxiety (λ = 0.093–0.677, *M* = 0.404) factors, showing that each item was loaded better on the general factor than on their respective specific factors. For the social interaction anxiety factor, five social interaction anxiety items (items 1, 2, 3, 4, and 5) showed a significant loading on the social interaction anxiety factor (λ ≥0.30), whereas one item (item 6) failed to load onto the factor (λ = 0.157). Five social performance anxiety items (items 7, 9, 10, 11, and 12) had a significant loading on the social performance anxiety factor (λ ≥0.30), whereas one social performance anxiety item (item 8) did not load onto the target factor (λ = –0.093). In other words, 10 of the 12 items exhibited significant factor loadings on their corresponding specific factors, suggesting that specific factor cross-loadings persisted in the bifactor ESEM model, even after accounting for the general factor.

**Table 4.** **Standardized factor loadings for the bifactor ESEM outcomes of the SIAS-6 and SPS-6**.

Item	General Factor	Factor I	Factor Ⅱ
1	**0.628**	**0.433**	0.043
2	**0.563**	**0.644**	0.020
3	**0.650**	**0.411**	0.049
4	**0.609**	**0.481**	–0.047
5	**0.587**	**0.539**	–0.036
6	**0.662**	**0.157**	–0.031
7	**0.723**	0.032	**0.323**
8	**0.905**	–0.050	**–0.093**
9	**0.680**	0.113	**0.349**
10	**0.668**	0.000	**0.408**
11	**0.635**	–0.022	**0.677**
12	**0.629**	–0.065	**0.571**
*ω*_H_	0.795		
*ω*_H⁢S_		0.206	0.202
ECV	0.681	0.168	0.151

Note: The target loadings are in bold font.

In the bifactor CFA and ESEM models, items related to social interaction anxiety and social performance anxiety loaded significantly onto both the general and specific factors, suggesting that their variances were divided into general and specific components. Consequently, the social interaction and performance anxiety factors were distinct from each other.

### 3.2 Reliability Assessments of the Bifactor Models

For the bifactor models, we computed various indices to assess reliability, including ω_H_, ω_H⁢S_, and the ECV. As shown in Table 3, ω_H_ for the general factor (12 items) was 0.795, while ω_H⁢S_ was 0.282 for the social interaction anxiety factor and 0.224 for the social performance anxiety factor. The general factor’s ω_H_ was above the acceptable cut-off score of 0.500, while the ω_H⁢S_ for the specific factors was moderate. The ECV for the general and social interaction anxiety factors were 0.682, 0.159, and 0.158, respectively. Thus, the general factors of the SIAS-3 and SPS-6 explained significantly more of the common variance than the two specific factors of the SIAS-3 and SPS-6 in the bifactor CFA model. However, because the ECV of the general factor did not exceed 0.70, the bifactor CFA model could not be considered unidimensional.

As shown in Table 4, the ω_H_ for the general factor (12 items) was 0.795, while the ω_H⁢S_ for the social interaction anxiety factor was 0.206 and for the performance anxiety factor was 0.202. The general factor’s ω_H_ exceeded the acceptable cut-off score of 0.500 and the ω_H⁢S_ of the specific factors was moderate. The ECV of the general and social interaction anxiety factors were 0.681, 0.168, and 0.151, respectively. Thus, the general factor of the SIAS-6 and SPS-6 explained approximately two-thirds of the common variance, while one-third of the common variance was accounted for by the two specific factors of the SIAS-6 and SPS-6 in the bifactor ESEM model. The estimated ECV for the general factor was below 0.70, indicating that the bifactor ESEM model could be considered multidimensional rather than unidimensional.

### 3.3 Measurement Invariance Analysis Across Genders

Following the factor analysis results, the measurement invariance analyses focused on the bifactor CFA and bifactor ESEM models, as these provided the best fit for the data. Measurement invariance was examined using multigroup analyses across genders. The findings of the measurement invariance analyses for the bifactor CFA model and for the bifactor ESEM model are shown in Table 5. As seen in the table, for bifactor CFA model, the changes in CFI and RMSEA from configural to metric were –0.004 and 0.001, respectively, which is suitable for invariance, considering Chen’s guidelines [30] for CFI (≤–0.010) and RMSEA (≥0.015) changes. The differences in RMSEA and CFI between metric to scholar invariance were ΔRMSEA = 0.002 and ΔCFI = –0.004 for bifactor CFA model. These differences were also considered invariant.

**Table 5.** **Fit indices for testing measurement invariance: Gender**.

Analysis	Model	CFI	RMSEA	90% CI
B-CFA	Configural	0.980	0.062	0.041–0.082
	Metric	0.976	0.061	0.041–0.078
	Scalar	0.972	0.063	0.045–0.079
B-ESEM	Configural	0.967	0.073	0.051–0.093
	Metric	0.987	0.038	0.000–0.060
	Scalar	0.980	0.045	0.019–0.065

Note: B-CFA, bifactor confirmatory factor analysis; B-ESEM, bifactor exploratory structural equation modeling.

Additionally, for the bifactor ESEM model, the changes in CFI and RMSEA from configural to metric were 0.020 and –0.035, respectively, which is suitable for invariance, considering Chen’s guidelines [30] for RMSEA (≥0.015) and CFI changes (≤–0.010). The changes in RMSEA and CFI from metric to scholar invariance were ΔRMSEA = 0.005 and ΔCFI = –0.007 for the bifactor ESEM model. These changes can also be considered as invariant. Thus, little change in model fit was observed between the different models, indicating overall measurement invariance across genders.

## 4. Discussion

The SIAS-6 and SPS-6 were originally designed to represent two different aspects of social anxiety, but previous studies have reported mixed findings regarding the dimensional structure of both scales. The current study sought to explore alternative models for the factor structure of the SIAS-6 and SPS-6 and to identify the most suitable model. Therefore, to establish the dimensional structure of the SIAS-6 and SPS-6, ESEM models were newly incorporated into the study, in addition to the models analyzed in previous research. To our knowledge, this study is the first to utilize ESEM to examine the factor structures of the SIAS-6 and SPS-6.

The findings showed that the bifactor CFA and ESEM models had a better fit than the one-factor, two-factor CFA, and two-factor ESEM models. In the bifactor models, the general factor exhibited better loadings, omega coefficients, and ECV scores than the two specific factors. However, social interaction anxiety and social performance anxiety items loaded significantly onto both the general and their designated specific factors in the bifactor models. Additionally, the omega coefficients of the specific factors and the ECV of the general factor indicate a multidimensional factor structure rather than a unidimensional factor structure.

The results of this study, favoring the bifactor model, could integrate different views of the relationship between social interaction and social performance anxiety. Findings favoring the bifactor model indicate that social interaction and social performance anxiety are closely related. Social interaction and social performance anxiety together constitute a general factor called global social anxiety, which is shared by the two aspects of social anxiety. This supports the idea that social interaction and social performance anxiety are conceptually related. However, the findings favoring the bifactor model also suggest that social interaction and social performance anxiety constitute specific factors with unique variances, even when controlling for the general factor of global social anxiety. These findings support the idea that social interaction and social performance anxiety are closely related concepts at the general factor level but are also distinct concepts.

The results of the bifactor ESEM and bifactor CFA were similar. This suggests the following. First, both the bifactor ESEM and CFA aim to model a general factor alongside several specific factors. The similarity in the results between the two models suggests that the identified factor structure with a general factor and two specific factors reflects a robust and meaningful underlying pattern in the data [19, 20]. Second, the bifactor ESEM allows greater flexibility by permitting cross-loadings, whereas the bifactor CFA typically restricts these cross-loadings. The similarity in the results between the models may indicate that each item predominantly aligns with one specific factor, or that each item is accurately capturing the intended construct [19, 20].

In both the bifactor ESEM and bifactor CFA, five of the six items for social interaction anxiety and five of the six items for social performance anxiety exhibited significant loadings onto their respective specific factors. Additionally, all items showed significant loadings for the general factors. Therefore, specific factors had little influence on item 6 (e.g., ‘I find it difficult to disagree with another’s point of view’), belonging to social interaction anxiety, and item 8 (e.g., ‘I worry about shaking or trembling when I am watched by other people’), belonging to social performance anxiety, when controlling for the influence of the general factor. Thus, while these two items, with only general factor loadings, can provide a good measure of overall social anxiety, they might lack the diagnostic precision to differentiate between the subtypes of social anxiety. Without this differentiation, it may be difficult to customize treatments or interventions to meet the specific needs of each individual.

This study has several limitations. First, different results may have been obtained if the study was conducted in a clinical setting. Therefore, there is a need to replicate the results of this study in individuals with social anxiety disorder in future studies. Second, data were collected only once. Thus, a longitudinal measurement invariance analysis is needed to assess the stability of the factor structures of the SIAS-6 and SPS-6. Third, this study used a self-reported questionnaire, and the results may have differed if an interview-type scale had been used. Therefore, there is a need to replicate the results of this study using an interview scale in future research. Fourth, in this study, the measurement invariance test by age or region could not be conducted because Chen [30] noted that smaller groups (e.g., fewer than 100 participants) might lead to unstable and unreliable results. Therefore, it is necessary to conduct measurement invariance tests by age or region using larger sample sizes in follow-up studies. Lastly, this study could not collect information on marital status, education level, socioeconomic status, etc., which may have affected social anxiety symptoms. Future studies should include this information and consider the impact of these variables on the results of this study.

## 5. Conclusion

In the present study, the bifactor models provided an integrated perspective on conflicting opinions regarding the relationship between social interaction and social performance anxiety. This is because the bifactor models simultaneously measure the global social anxiety shared by social interaction and social performance anxiety, as well as the specific factors of each. If a bifactor model is applied in future research, it will be possible to determine how global social anxiety and the specific factors of social interaction and social performance anxiety predict and relate to other variables.

## Availability of Data and Materials

All data generated or analyzed in this study are available from the corresponding author upon reasonable request.
